# Unveiling the Genetic Association Between Hemoglobin Concentration and Amyotrophic Lateral Sclerosis

**DOI:** 10.1002/brb3.71152

**Published:** 2025-12-31

**Authors:** Hongmei Luo, Yujie Yang, Xiaojian Cao, Chunchu Deng, Hong Chen

**Affiliations:** ^1^ Department of Rehabilitation, Tongji Hospital, Tongji Medical College Huazhong University of Science and Technology Wuhan China; ^2^ Stem Cell Research Center, Tongji Hospital, Tongji Medical College Huazhong University of Science and Technology Wuhan China; ^3^ Hubei Key Laboratory of Neural Injury and Functional Reconstruction Huazhong University of Science and Technology Wuhan China

**Keywords:** amyotrophic lateral sclerosis, hemoglobin concentration, induced pluripotent stem cell, Mendelian randomization

## Abstract

**Background:**

Although previous studies have suggested an association between hemoglobin (Hb) concentration and amyotrophic lateral sclerosis (ALS), the precise cause‐and‐effect relationship between them is still unclear. This study aims to investigate the causal correlation between Hb concentration and ALS, and explore the potential genes related to their association.

**Methods:**

We extracted summary statistical data of Hb concentration and ALS from genome‐wide association studies (GWAS), performed Mendelian randomization (MR) analyses, and conducted RNA sequencing of motor neurons different from ALS patient‐derived induced pluripotent stem cells (iPSCs), followed by an intersection analysis between differentially expressed genes (DEGs) in ALS motor neurons and selected instrumental variables (IVs) associated with Hb concentration.

**Results:**

As a result, Hb concentration had a negative causal relationship with the risk of ALS, established through IVW (OR = 0.854; 95% CI: 0.767–0.951; *p* = 0.00418) of the univariable MR analysis. A multivariable MR further confirmed that this causal link remained robust, even when accounting for confounders including systolic blood pressure, total cholesterol levels, body mass index, LDL cholesterol, diastolic blood pressure, and smoking. Importantly, genetically predicted ALS did not show a causal connection to Hb concentration. Additionally, RNA sequencing analysis and qRT‐PCR results revealed that transcripts for *BACH1* and *FLVCR1* were upregulated, while those for *TRIM58* were downregulated in *SOD1* ^D90A^ ALS motor neurons, compared to the control. In motor neurons differentiated from a sporadic ALS patient‐derived iPSCs, qRT‐PCR showed increased transcript levels of *BACH1*, and decreased transcript levels of *FLVCR1* and *TRIM58*. These three genes were intersected with harmonized SNPs between Hb concentration and ALS.

**Conclusion:**

Our study concludes that genetically predicted Hb concentration exhibited an independent inverse causal association with the risk of developing ALS, with potential involvement of genes such as *BACH1*, *FLVCR1*, and *TRIM58*.

## Graphical abstract

This study identified a causal relationship between genetically predicted hemoglobin (Hb) concentration and amyotrophic lateral sclerosis (ALS) through Mendelian randomization (MR), and integrated RNA‐seq and RT‐qPCR analyses to highlight *BACH1*, *FLVCR1*, and *TRIM58* as potential genes involved in this association.

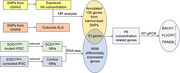

## 1 Introduction

ALS, as a fatal adult‐onset disease, is marked by the degeneration of upper and lower motor neurons (Rowland and Shneider 2001; Deng and Chen 2024). This degeneration leads to progressive voluntary muscle weakness and atrophy, ultimately resulting in death in most cases within 2 to 5 years of onset, primarily due to respiratory failure (Rowland and Shneider 2001). About 5–10% of ALS cases are familial ALS (fALS), primarily resulting from mutations in certain genes such as *SOD1*, *FUS*, *TARDBP* (TDP‐43), *VAPB*, *MATR3* (matrin‐3), and *ATXN2* (ataxin‐2) (Yousefian‐Jazi et al. 2020; Neupane, Thada et al. 2023). The other cases are sporadic ALS (sALS), which arise without any known causes or a family history (Yousefian‐Jazi et al. 2020; Neupane, Thada et al. 2023). Riluzole, Edaravone, Relyvrio (AMX0035), and Tofersen are medications approved by the FDA for ALS, yet their effectiveness seems to be restricted (Neupane, Thada et al. 2023; Ansari, Alam et al. 2024; Wiesenfarth et al. 2024).

Previous studies have identified several risk factors potentially linked to ALS. Recent findings suggest that levels of Hb may be associated with incidences of neurodegenerative diseases, including ALS (Abbott et al. 2012; Mandrioli, Rosi et al. 2017). Hb is an iron‐rich protein that plays a crucial role in transporting oxygen within the bloodstream (Gell 2018). A recent label‐free quantitative proteomics study of ten patients with sALS revealed that the serum levels of hemoglobin beta‐1 (HBB1) increased as the disease progressed (He, Zhou et al. 2024). Additionally, hemoglobin subunit alpha (HBA1) levels were elevated in the serum of early‐stage sALS patients compared to healthy controls (He, Zhou et al. 2024). However, a retrospective study involving 275 ALS patients indicated that higher Hb levels were inversely associated with increased odds of death or the need for tracheostomy (Mandrioli, Rosi et al. 2017). Other studies have also shown that ALS patients generally exhibit lower levels of Hb, transferrin, albumin, and prealbumin (Chełstowska and Kuźma‐Kozakiewicz 2020; Janse van Mantgem et al. 2020). High‐caloric nutrition significantly improves the cumulative survival rate in ALS patients (Wang et al. 2022). In those receiving high‐caloric nutrition, levels of Hb, body weight, transferrin, albumin, and prealbumin were notably elevated (Wang et al. 2022). These findings suggest that lower levels of Hb could be linked to a higher risk of ALS.

The complex relationship between Hb and ALS may be influenced by reverse causality, confounding factors, and other biases, making it difficult to establish causal inferences in observational studies (Ejima, Li et al. 2016). Previous research has demonstrated associations between Hb levels and various factors, such as blood pressure, low‐density lipoprotein cholesterol (LDL‐C), total cholesterol levels, body mass index (BMI), and smoking status, concerning ALS (Hämäläinen et al. 2018; Pedersen et al. 2019; Kamruzzaman 2021; Xia, Zhang et al. 2022; Li, Wei et al. 2023; Schneekloth et al. 2024; Zhu et al. 2025). As a result, it is still unclear whether Hb levels are independently associated with the risk of developing ALS or if the other factors contribute to the observed relationship between Hb concentration and ALS. MR leverages single‐nucleotide polymorphisms (SNPs) as IVs to elucidate causal relationships between exposures and outcomes (Skrivankova et al. 2021). This approach minimizes the influence of lifestyle and environmental variables, mitigating biases associated with unobserved confounding and reverse causation. By utilizing the genetic variation inherent in SNPs, MR enhances the robustness of causal inference (Skrivankova et al. 2021).

In this study, we utilized summary‐level data from GWAS focused on Hb concentration and ALS in populations of European ancestry to estimate the causal effects between these two variables using bidirectional and univariable MR (UVMR) analyses. Subsequently, a multivariable MR (MVMR) study was conducted to account for various confounding factors, including blood pressure, LDL‐C, total cholesterol levels, BMI, and smoking. Following this, we conducted RNA sequencing on motor neurons differentiated from induced pluripotent stem cells (iPSCs) derived from ALS patients with the *SOD1* ^D90A^ mutation. We used genetically corrected *SOD1* ^D90A^ iPSCs as the control. We then analyzed the correlation between differentially expressed genes (DEGs) and harmonized SNPs between Hb concentration and ALS. Finally, we performed quantitative reverse transcription polymerase chain reaction (qRT‐PCR) to validate the alterations in Hb‐related genes in *SOD1* ^D90A^ ALS and sALS motor neurons. This allowed us to identify Hb‐related genes that are altered in ALS. By identifying potential risks and protective factors for ALS, this study aims to enhance our understanding of the disease and aid in developing more effective treatment strategies.

## 2 Methods

### 2.1 Overall Study Design

**FIGURE 1:**
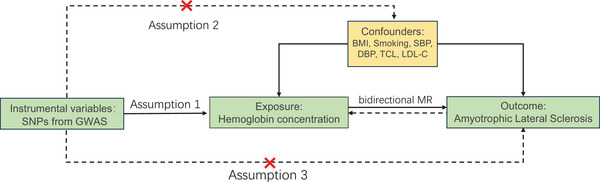
Flowchart illustrating the MR study design for investigating the relationship between hemoglobin concentration and ALS. SNPs, single nucleotide polymorphisms; BMI, body mass index; SBP, systolic blood pressure; DBP, diastolic blood pressure; TCL, total cholesterol levels; LDL‐C, LDL Cholesterol.

The methodology of our MR analysis is illustrated in Figure 1. The genetic variants employed in this study were selected based on three essential assumptions that underpin robust causal inference (Davies, Holmes et al. 2018): (i) a significant association between the genetic variants and the exposure variables; (ii) an absence of correlation between these genetic variants and any potential confounders; and (iii) the premise that genetic variants exert their effects on outcomes solely via the exposure pathway. We evaluated the causal relationship between Hb concentration and ALS through a bidirectional UVMR analysis. Subsequently, MVMR analyses were conducted to verify the independence of this causal link while controlling confounding variables. All genetic data utilized in this MR study were obtained from the IEU Open GWAS project, with all ethical approvals duly noted in the original GWAS publications.

### 2.2 Data Source

The genetic exposure data for Hb concentration was sourced from the UK Biobank, specifically from Mbatchou J's lab (GWAS ID: ebi‐a‐GCST90013978). This dataset comprises 10,783,698 SNPs derived from a total of 350,474 individuals. The genetic data for ALS was collected from a GWAS conducted by Nicolas et al. (GWAS ID: ebi‐a‐GCST005647), which includes 20,806 ALS cases compared to 59,804 controls. All participants in these studies are of European descent. In our MVMR analysis, we incorporated five confounding variables based on prior research findings (Greco, Minelli et al. 2015; Burgess, Bowden et al. 2017; Burgess and Thompson 2017; Chang et al. 2022; Zhou, Jiang et al. 2024). These include smoking (GWAS ID: ukb‐b‐20261) and body mass index (BMI) (GWAS ID: ukb‐b‐19953) from the UK Biobank, systolic blood pressure (GWAS ID: ebi‐a‐GCST90000062), diastolic blood pressure (GWAS ID: ebi‐a‐GCST90000063), total cholesterol levels (GWAS ID: ebi‐a‐GCST90025953), and LDL‐C (GWAS ID: ebi‐a‐GCST90018961). During the harmonization process, we excluded palindromic SNPs with intermediate allele frequencies.

### 2.3 Selection of Instrumental Variables

To derive strong IVs for Hb concentration, we selected independent SNPs exhibiting genome‐wide significance (*p* < 5×10^−8^) for MR analysis. We then implemented a clumping procedure (*r* ^2^ < 0.001, kb = 10,000) to address linkage disequilibrium (LD), using a clumping window of 10,000 base pairs grounded in the European 1000 Genomes Project reference panel. Additionally, we established that the F‐statistic must exceed 10 to mitigate weak instrument bias. The F‐statistic was calculated using the formula *F* = *R* ^2^ × (*N* − 2) / (1 − *R* ^2^), where *R* ^2^ reflects the proportion of variance accounted for by the selected SNPs in the exposure dataset. *R* ^2^ was derived from the equation *R* ^2^ = 2 × EAF × (1 − EAF) × *β* ^2^, with EAF denoting the effect allele frequency and *β* representing the effect size of the allele.

### 2.4 Statistical Analysis

This MR analysis was conducted using the TwoSampleMR package in R version 4.4.1, along with the MR‐PRESSO package. We primarily utilized the inverse variance weighted (IVW) method, which is one of the five MR analysis approaches, to evaluate the causal relationship between the exposure and the outcome (Burgess et al. 2019). In order to evaluate the reliability of our results, we conducted sensitivity analyses that incorporated tests for horizontal pleiotropy and heterogeneity, together with the weighted median (WM) method (Chang et al. 2022; Zhou, Jiang et al. 2024). We assessed heterogeneity among the IVs using Cochran's Q, along with both the MR Egger method and the IVW method (Burgess, Bowden et al. 2017). Furthermore, we conducted the MR Egger regression intercept test and the MR‐PRESSO global test to examine horizontal pleiotropy (Greco, Minelli et al. 2015). Additionally, we performed a leave‐one‐out sensitivity analysis to investigate the impact of each SNP on the overall MR results by stepwise removal of SNPs (Burgess and Thompson 2017). Statistical significance was defined as *p* < 0.05.

### 2.5 RNA‐seq Library Construction and Sequencing of Motor Neurons Differentiated From iPSCs Derived From ALS Patients

Three iPSC lines used in this study were kindly provided by Professor Suchun Zhang's laboratory (Chen et al. 2014). The first line was derived from an ALS patient carrying the *SOD1* ^D90A^ mutation, the second from a sALS patient, and the third was a gene‐corrected isogenic control line, in which the D90A mutation was precisely corrected to wild‐type residue (Control) using Transcription Activator‐Like Effector Nuclease (TALEN)‐mediated genome editing (Chen et al. 2014). The protocol for differentiating iPSCs into motor neurons was adapted and modified based on a previously published study by the Chen laboratory (Du et al. 2015). Specifically, iPSCs were cultured on a feeder layer of mouse embryonic fibroblasts using a maintenance medium composed of DMEM/F12, 20% KSR, 1% (v/v) NEAA and GlutaMax, 0.1 µM β‐mercaptoethanol, and 8 ng/mL bFGF. IPSC colonies were digested with dispase and plated onto 6‐well plates containing a fresh feeder layer. On the second day, the maintenance medium was replaced with a differentiation medium. The differentiation medium consisted of basal medium and small molecules. The basal medium included DMEM/F12 and Neurobasal in a 1:1 ratio, supplemented with 1% (v/v) GlutaMax, 1% B27, 0.5% N‐2, and 0.1 mM ascorbic acid. Small molecules were added to promote differentiation toward spinal motor neurons, including 3 µM CHIR, 2 µM DMH1, and 2 µM SB. The medium was refreshed every two days.

After 7 days of differentiation, the CHIR concentration was reduced to 1 µM, and 0.1 µM RA and 1 µM SAG were added to further differentiate the cells for an additional 7 days, resulting in motor neuron progenitor cells. The cells were then subjected to suspension culture with 0.01 µM RA and 0.1 µM SAG in the basal medium for another 7 days. The suspended neurospheres were digested into smaller neurospheres using Accutase and plated onto glass coverslips pre‐coated with Matrigel. Neurobasal medium supplemented with 0.1 µM Compound E was used to synchronize motor neuron maturation for 3 days, followed by the addition of 10 ng/mL IGF‐1, 10 ng/mL BDNF, and 10 ng/mL GDNF. The cells were cultured for 10 more days to obtain mature motor neurons (MNs) expressing CHAT^+^.

The RNA‐seq library was constructed with VAHTS Universal V10 RNA‐seq Library Prep Kit (Vazyme, NR616‐01) following the manufacturer's instructions.

### 2.6 Processing of RNA‐seq Data and Visualization

The raw RNA‐seq data was first trimmed to remove adaptor sequences and then quality‐checked using FastQC. (http://www.bioinformatics.babraham.ac.uk/projects/fastqc). The alignment index was downloaded from http://daehwankimlab.github.io/hisat2/download/#h‐sapiens, and the gtf file was downloaded from ENSEMBL (https://ftp.ensembl.org/pub/release‐113/gtf/homo_sapiens/). The pre‐processing of RNA‐seq data was conducted by a pipeline provided by Vivek Thakur (Thakur 2024). The differential analysis was conducted by the R package DESeq2 (Love et al. 2014). The visualization of MR analysis data was conducted using by built‐in functions of the R package TwoSampleMR (Hemani et al. 2018). The volcano plot was conducted by the R package EnhancedVolcano (https://github.com/kevinblighe/EnhancedVolcano). The expression of genes was visualized by GraphPad Prism, normalized count corresponding to every sample was used.

### 2.7 Immunofluorescence Staining

After 10 days in culture on Matrigel‐coated slides, motor neurons were fixed in 4% paraformaldehyde (PFA) at room temperature for 15 min. After fixation, the cells were permeabilized and incubated with a Blocking Buffer that included Triton X‐100 (Beyotime, P0260) for 30 min at room temperature. Primary antibodies (MAP2, Sigma, M1406; CHAT, Millipore, ab144P) were subsequently administered and allowed to incubate overnight at 4°C. The next day, the motor neurons underwent three washes with PBS, followed by a 1‐h incubation with secondary antibodies. Afterward, the cells were treated with Hoechst stain for a duration of 8 min. After three additional washes, Aqua Poly/Mount (Polysciences, 18606–20) was used for mounting the samples.

### 2.8 Quantitative Reverse Transcription Polymerase Chain Reaction (qRT‐PCR)

Total RNA was isolated from cells using the TRIzol Reagent (Invitrogen, USA) following the manufacturer's guidelines. The purity and concentration of the extracted RNA were evaluated using a NanoDrop spectrophotometer (Thermo Fisher Scientific, USA). Subsequently, 1 µg of RNA was used for complementary DNA (cDNA) synthesis with the PrimeScript RT Reagent Kit (Takara, Japan), according to the manufacturer's instructions.

qRT‐PCR was conducted on a QuantStudio 5 Real‐Time PCR System (Applied Biosystems, USA) utilizing the TB Green Premix Ex Taq II (Takara, Japan). Each 20 µL reaction mixture consisted of 2 µL of cDNA, 10 µL of TB Green Premix, 0.4 µL of each primer (10 µM), and 7.2 µL of nuclease‐free water. The PCR program included an initial denaturation at 95°C for 30 s, followed by 40 amplification cycles of 95°C for 5 s and 60°C for 30 s.

The relative expression of target genes was quantified using the 2^−ΔΔCt^ method, with GAPDH serving as the internal reference for normalization. Primer sequences for qRT‐PCR are provided as follows: GAPDH‐F: ATCGTGGAAGGACTCATGACC; GAPDH‐R: AGGGATGATGTTCTGGAGAGC; BACH1‐F: CGCCTCAGCTCTGGTTGAT; BACH1‐R: CATCAGCCTGGCCTACGATT; FLVCR1‐F: AGGGGTGCTTGGCTTCTTCA; FLVCR1‐R: AATGTTCCCTGCCTTAGGACC; TRIM58‐F: CGGCAGCTACCAGGTAAAGC; TRIM58‐R: GCTGCCTCTGCATTTCCACT. Each reaction was performed in triplicate to ensure reproducibility.

## 3 Results

### 3.1 Genetically Predicted Hemoglobin Concentration Is Causally Linked to the Risk of Amyotrophic Lateral Sclerosis

This MR study was conducted in accordance with the Strengthening the Reporting of Observational Studies in Epidemiology (STROBE)‐MR guidelines (Table S1). In this study, we identified 364 SNPs associated with hemoglobin concentration, achieving a significance threshold of *p* < 5 × 10^−8^ based on our IVs selection criteria. After removing SNPs that were correlated with potential confounders, 361 SNPs remained. The subsequent harmonization process led to the exclusion of two SNPs, resulting in a final dataset comprising 304 SNPs for analysis (Table S2). The F‐statistics for the included SNPs varied from 29.793 to 1473.724, indicating a strong robustness for the IVs employed.

**FIGURE 2:**
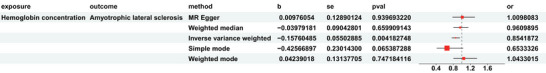
Forest plot demonstrating the results of the UVMR study on the causal link between hemoglobin concentration and ALS. OR, odds ratio; b, effect size; se, standard error of the effect size; IVW, inverse variance weighted. A *p*‐value < 0.05 was considered statistically significant. OR > 1 indicates a risk factor, while OR < 1 suggests a protective factor.

**FIGURE 3:**
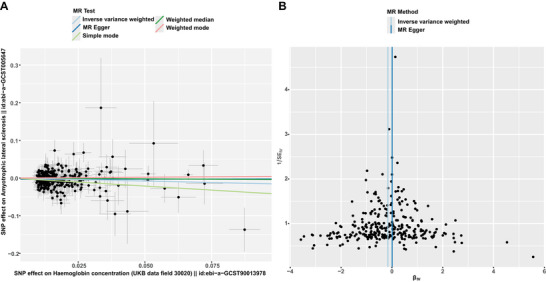
Results from the UVMR analyses examining the causal relationship between hemoglobin concentration and ALS. (A) Scatter plot depicting the causal relationships between hemoglobin concentration and ALS, with slopes representing the estimated causal effect magnitude. (B) Funnel plot illustrating heterogeneity tests, highlighting causal effect estimates for hemoglobin concentration and ALS. Lines represent causal effect estimates derived from the inverse variance weighting and MR‐Egger models.

The results from the IVW analysis in our UVMR study indicate an inverse causal relationship between Hb concentration and the risk of ALS, with an odds ratio (OR) of 0.854 (95% CI: 0.767–0.951; *p* = 0.00418), as illustrated in Figure 2. Consistency in the direction of effect was confirmed through both the WM and simple mode methods employed in the UVMR. The causal effect estimates are visually represented in scatter plots (Figure 3A). Cochran's Q test for heterogeneity revealed no significant variance in the causal estimates from both MR Egger and IVW analyses (*p* > 0.05). Furthermore, the MR‐Egger intercept showed no significant pleiotropy (–0.0044; *p* = 0.076), a finding corroborated by the MR‐PRESSO global test (*p* = 0.66) as detailed in Table 1. The symmetry observed in the funnel plot around the null effect further supports the absence of horizontal pleiotropy (Figure 3B).

**TABLE 1:** Sensitivity analysis for the causal effect of Hb concentration on ALS.

Exposure	Outcome	Heterogeneity		Pleiotropy		Weighted median
		Cochran's *Q*		Egger Intercept *P*	MR‐PRESSO global test *P*	*p*
		MR Egger *P*	the IVW *P*			
Hemoglobin concentration	Amyotrophic lateral sclerosis	0.784	0.77	0.076	0.66	0.66

**FIGURE 4:**
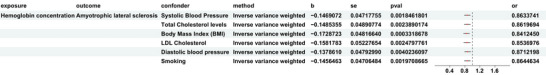
Forest plot demonstrating the results of the MVMR study on the causal effects of hemoglobin concentration on ALS. OR, odds ratio; b, effect size; se, standard error of the effect size; IVW, inverse variance weighted: *p* < 0.05 was considered significant. OR>1 is considered as a risk factor, and OR<1 is considered as a protective factor.

Leave‐one‐out sensitivity analyses confirmed that no individual single SNP had a disproportionate effect on the overall causal estimate linking Hb concentration to ALS (Figure S1). Additionally, we explored the potential for reverse causality where ALS acts as the exposure and Hb concentration as the outcome; our analysis revealed no evidence that ALS is a genetically causal factor for Hb concentration (OR = 0.994; 95% CI: 0.935–1.057; *p* = 0.859) (Figure S2). To account for potential confounding variables, an MVMR analysis was conducted, the summary of which is presented in Figure 4. The findings affirm that the relationship between Hb concentration and ALS risk remains robust when adjusted for possible confounders.

### 3.2 Hemoglobin‐Related Transcripts Are Differentially Expressed in Motor Neurons Differentiated From ALS Patient‐Derived iPSCs

**FIGURE 5:**
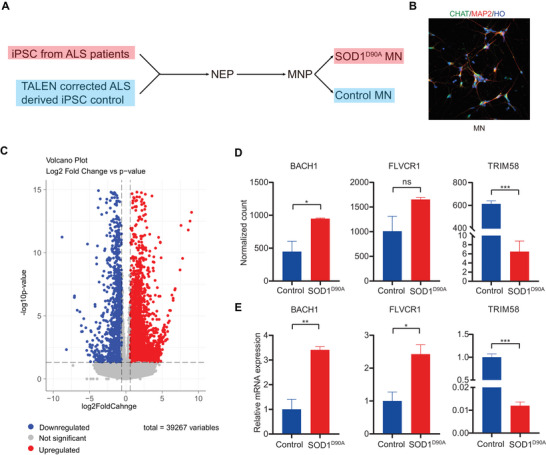
Analysis of RNA sequencing data from motor neurons with ALS *SOD1* D90A mutation and genetically corrected controls. (A) Schematic plot of motor neuron differentiation from iPSCs derived from an ALS patient with the D90A mutation. Using TALEN technology, genetically corrected D90A mutation iPSCs were used as a control. (B) Immuno‐fluorescence plot of mature MN, with green indicating CHAT, red indicating MAP2, and blue indicating Hoechst. (C) Volcano plot illustrates the significantly upregulated (red) and downregulated genes between disease motor neurons and control cells (blue), while gray dots indicate genes without significant change over transcript level between groups. (D) Bar plot indicating normalized count between two groups of *BACH1*, *FLVCR1*, and *TRIM58* genes, with error bars representing the mean ± standard deviation (SD). (E) qRT‐PCR analysis showing the expression level of *BACH1*, *FLVCR1*, and *TRIM58* genes between two groups, with error bars representing the mean ± SEM. Statistical significance is denoted by asterisks: *p < 0.05 and ***p < 0.001, while ns represents not significant. Asterisks indicate significant differences between groups as determined by the *t*‐test.

Using a differentiation protocol previously published by our group (Chen et al. 2014), we conducted RNA sequencing on motor neurons differentiated from human iPSCs carrying the *SOD1* ^D90A^ mutation and their gene‐corrected isogenic controls in order to investigate the transcriptional correlation between Hb concentration and ALS. The process of motor neuron differentiation obtained from ALS patients was shown in Figure 5A, and the motor neurons were validated by immunofluorescence staining (Figure 5B). In the transcript spectrum comparison analysis, 4565 upregulated genes and 2331 downregulated genes could be identified after filtering DEGs based on *p*‐value (<0.05) and log_2_foldchange (←log_2_1.5 or >‐log_2_1.5) (Figure 5C). To investigate the correlation between the harmonized IVs associated with Hb concentration and ALS, and genes potentially implicated in ALS, we annotated the SNP accession numbers of the selected IVs to their corresponding genes and performed intersection analysis with the DEGs list. The analysis revealed that *BACH1* and *FLVCR1* exhibited significantly elevated expression levels, whereas *TRIM58* demonstrated significantly decreased expression levels in *SOD1* ^D90A^ mutant cell lines (Figure 5D). Further qRT‐PCR analysis confirmed the increased expression levels of *BACH1* and *FLVCR1* transcripts, alongside a decrease in the expression levels of *TRIM58* transcripts in ALS *SOD1* ^D90A^ mutant motor neurons (Figure 5E). To further access the generalizability of these findings, we evaluated transcript levels of *BACH1*, *FLVCR1*, and *TRIM58* in motor neurons derived from a sALS patient (Figure S3). Consistent with our results in the *SOD1* ^D90A^ model, *BACH1* expression was elevated and *RIM58* expression was reduced compared with the control. However, unlike the pattern observed in the *SOD1* ^D90A^ line, *FLVCR1* expression was decreased in sALS‐derived motor neurons. This divergence suggests that while *BACH1* and *TRIM58* show more consistent dysregulation across distinct ALS backgrounds, *FLVCR1* expression may be more sensitive to underlying genetic heterogeneity or represent subtype‐specific transcriptional alterations.

The data suggests that the three genes *BACH1, FLVCR1*, and *TRIM58* may play a significant role in the causal relationship between Hb concentration and ALS. Nonetheless, confirming their generalizability will require additional stratified analyses across ALS subgroups with diverse genetic backgrounds, including cases carrying mutations in genes such as *SOD1*, *FUS*, *TARDBP*, *VAPB*, *MATR3*, or *ATXN2*.

## 4 Discussion

Using MR analysis, we estimated the causal link between Hb and ALS. Initially, our results demonstrated that increased levels of Hb have an adverse impact on the progression of ALS, while ALS is not causally linked to Hb levels, indicating that higher Hb levels are protective for ALS development. Moreover, the findings from the multivariable MR analysis revealed that confounding variables such as blood pressure, LDL‐C, total cholesterol levels, BMI, and smoking do not play a role in the causal relationships between Hb and ALS, despite the connections of these factors to both Hb and ALS indicated in previous studies (Hämäläinen et al. 2018; Pedersen et al. 2019; Kamruzzaman 2021; Xia, Zhang et al. 2022; Li, Wei et al. 2023; Schneekloth et al. 2024; Zhu et al. 2025). Our RNA sequencing analysis demonstrated that expression levels of transcripts of *BACH1* and *FLVCR1*, associated with Hb, are significantly increased, while the expression of *TRIM58* was found to be decreased in ALS patients with the *SOD1* ^D90A^ mutation‐derived iPSC differentiated motor neurons. The identified three genes were analyzed in conjunction with the harmonized IVs that relate Hb concentration to ALS. Subsequent qRT‐PCR analysis validated the differential expression of transcripts for *BACH1, FLVCR1*, and *TRIM58* in motor neurons carrying the *SOD1* ^D90A^ ALS mutation.

The relationship between Hb levels and ALS risk or disease progression remains inconclusive across current studies. Although alterations in systemic iron metabolism have been repeatedly observed in ALS, direct and independent associations between Hb levels and disease risk or prognosis have not been consistently demonstrated. In a retrospective cohort of 275 ALS patients, higher Hb and hematocrit levels were associated with increased odds of death or tracheostomy (odds ratio for Hb = 1.71; 95% CI: 1.24–2.36); however, given its observational design, this study cannot establish causality (Mandrioli, Rosi et al. 2017). Despite adjustments for covariates, residual confounding from unmeasured factors—such as hydration status, nutritional support, or disease heterogeneity—may have influenced the observed associations. The cohort size (*n* = 275) and single‐center design limit the statistical power and external validity of findings (Mandrioli, Rosi et al. 2017).

In a large prospective Chinese cohort (*n* = 450), elevated glycated hemoglobin (HbA1c ≥ 6.5%)—but not fasting glucose—was significantly associated with increased mortality (adjusted HR = 2.06; 95% CI: 1.07–3.96), whereas standard Hb was not assessed, suggesting that chronic glycemic dysregulation may modulate ALS progression (Wei, Chen et al. 2017). Similarly, a Swedish population‐based cohort of 399 newly diagnosed ALS patients found no independent association between Hb levels and mortality after multivariable adjustment (Sun, Carrero et al. 2020). However, this study was conducted within a single regional healthcare system, which may limit generalizability to ethnically or environmentally diverse populations (Sun, Carrero et al. 2020). Meta‐analytic evidence supports the presence of iron dyshomeostasis in ALS, characterized by elevated serum ferritin and reduced transferrin concentrations compared with controls, while total serum iron and Hb levels remain largely unchanged (Wang, Li et al. 2020). Similarly, the large meta‐analysis by Cheng, Chen et al. (2021) found that elevated ferritin, transferrin saturation, and creatine kinase—rather than HbA1c—were linked to ALS or poorer survival, highlighting that disruptions in iron regulation and muscle metabolism may reflect disease pathophysiology. In a recent longitudinal analysis, Psychogios, Hu et al. (2024) reported that red blood cell–related indices, including increased levels of mean corpuscular Hb and mean corpuscular volume, were linked to higher mortality risk, suggesting that hematologic alterations may mirror systemic oxidative or metabolic stress in ALS (Psychogios, Hu et al. 2024). After multivariable adjustment, Hb remained associated with mortality at all time points, although the effect attenuated over time—hazard ratio (HR) = 2.07 (95% CI: 1.28–3.37) at 6 months, HR = 1.45 (95% CI: 1.06–1.97) at 1 year, and HR = 1.20 (95% CI: 0.99–1.46) at 3 years (Psychogios, Hu et al. 2024). In that analysis, Hb, erythrocyte volume fraction, and erythrocyte count—all components of the composite “Red Blood Cell Profile”—were independently associated with mortality, highlighting their potential utility as adjunct prognostic biomarkers (Psychogios, Hu et al. 2024). Nonetheless, metabolic and inflammatory parameters, such as creatinine, albumin, C‐reactive protein, and glucose, appear to be predictors of ALS survival (Psychogios, Hu et al. 2024). Collectively, these findings suggest that while alterations in Hb and related hematologic indices may reflect systemic physiological stress, this evidence did not support a direct causal or independent prognostic role of Hb in ALS. However, technical and methodological differences—such as variable laboratory assays, inconsistent definitions (e.g., Hb vs. hematocrit vs. ferritin), and differing statistical models—further limit cross‐study comparability. The relatively small sample sizes of many cohorts also constrain the power to explore effect modification by sex, site of onset, or genetic subtype. Therefore, while these studies collectively support systemic metabolic and iron‐related dysregulation in ALS, extrapolation to diverse global populations should be approached cautiously.

Our findings support a negative causal association between genetically predicted Hb concentration and the risk of ALS. Specifically, both univariable and multivariable MR analyses revealed that higher genetically determined Hb levels are associated with a reduced risk of developing ALS, independent of major confounders such as blood pressure, lipid profile, body mass index, and smoking. This observation aligns with previous epidemiological studies suggesting that lower peripheral Hb or hematocrit levels are linked to faster functional decline and poorer survival in ALS patients (Mandrioli, Rosi et al. 2017). Our MR analysis extends these observations by suggesting that genetically predicted Hb concentration exerts a causal protective effect against ALS susceptibility.

Our MR study has several advantages. First, we implemented a strict *p*‐value threshold of 5E‐8 for IVs selection to effectively minimize the weak instrument bias that could skew the overall estimates. Second, we incorporated summary‐level data from GWAS that included 4159 cases and 18,650 controls for ALS, as well as a sample size of 350,474 for Hb concentrations. These large sample sizes ensured sufficient statistical power to identify SNPs associated with both Hb levels and ALS risk, leading to robust causal estimates. Additionally, our study utilized a bidirectional approach to rule out the possibility of ALS causing changes in Hb levels. We also accounted for confounding factors in the multivariable MR analysis to mitigate their potential influence on the causal relationships between Hb and ALS. We must also recognize the limitations inherent in this MR study. Firstly, these results are derived from GWAS summary statistics of individuals of European ancestry, raising an important question about the generalizability of these findings to other ethnic groups. Secondly, the generalizability of our findings is limited by the use of pluripotent stem cell models derived from a *SOD1*‐mutant ALS patient line and a sALS patient line. We did not conduct stratified analyses across subgroups with more distinct genetic backgrounds, such as cases carrying mutations in genes including *SOD1*, *FUS*, *TARDBP*, *VAPB*, *MATR3*, or *ATXN2*. Genetic heterogeneity—such as differences in *C9orf72* repeat expansion frequency—as well as population‐specific variations in metabolic or nutritional status, may modulate hematologic biomarkers and alter their relationship with ALS susceptibility or progression. These factors collectively limit the external validity of our results. Replication in diverse genetic backgrounds and in more sALS models will be essential to confirm the robustness and translational relevance of these findings. Thirdly, we did not account for the disease stage among ALS cases. Research has indicated that although Hb levels were not significant initially in a dynamic prognostic and predictive model for ALS, follow‐up assessments showed that elevated Hb levels acted as a risk factor for ALS, with a risk estimate of 2.221 for every 10 g/L increase (Huang, Geng et al. 2023); thus, the relationship between Hb and ALS may differ across various stages of the disease.

The exact ways in which Hb influences the progression of ALS remain unclear. Hb consists of two sets of chains: one set of α‐like chains and another set of β‐like chains, each linked to a heme group. This heme comprises a tetrapyrrole structure (protoporphyrin IX) that contains a central ferrous ion (Fe^2^⁺), enabling it to reversibly bind to O_2_ and thus assisting in the transportation of oxygen from the lungs to the tissues of the body (Jorge, Ribeiro et al. 2016). *BACH1* is a transcription factor mainly tasked with overseeing the regulation of Hb synthesis expression (Belcher et al. 2023). It influences heme metabolism and oxygen transport functions by suppressing the transcription of genes related to heme and Hb (Belcher et al. 2023). Research has indicated that a specific *BACH1* inhibitor, ASP8731, can boost the levels of circulating fetal Hb cells, which may provide potential treatment for sickle cell anemia (Belcher et al. 2023). According to genes identified in our transcriptomic sequencing findings, *BACH1* is elevated in ALS D90A motor neurons, underscoring the connection between Hb and ALS. Feline leukemia virus subgroup C receptor 1 (*FLVCR1*) functions as a heme exporter in the cell membrane, crucial for maintaining the equilibrium between intracellular heme concentrations and globin synthesis in erythroid progenitors (Chiabrando et al. 2012). Heme production occurs within mitochondria, while globin synthesis occurs in the cytosolic compartment (Chiabrando et al. 2012; Fleming and Hamza 2012). *FLVCR1* is primarily responsible for facilitating heme export from the cytosol to the extracellular space, while the isoform FLVCR1b specifically mediates the translocation of heme from mitochondria to the cytosol (Chiabrando et al. 2012). Recent research suggests that a decrease in Hb within the mitochondria of neurons and glial cells could be linked to neurodegenerative disorders (Shephard, Greville‐Heygate et al. 2014; Vanni et al. 2018; Killinger et al. 2022). It is noteworthy that the expression of *FLVCR1* is increased in the D90A variant, possibly resulting in greater heme export from mitochondria or intracellular compartments, which leads to reduced levels of heme and Hb in these organelles. *TRIM58* is an E3 ubiquitin ligase that is recognized for its significant role in the development of red blood cells, known as erythropoiesis. Its involvement in erythropoiesis pertains to the regulation of Hb synthesis, mainly by affecting the structure and function of red blood cells. Existing literature suggests that *TRIM58* might play a part in the process of Hb switching (Movahedi Motlagh et al. 2023), although research on this gene and its relationship with Hb is still limited. It remains uncertain whether *TRIM58* is implicated in the causal relationships between Hb and ALS.

Hb in red blood cells can interact with metal ions and form complexes with both iron and zinc ions (Kosmachevskaya, Novikova et al. 2024). Disrupted iron homeostasis may influence the oxygen‐binding capacity of Hb, potentially leading to chronic hypoxia. Genetic mutations in the *SOD1* gene are considered risk factors for ALS. The presence of *SOD1* inclusions is a defining feature of ALS pathology. A lack of Cu^2+^ and Zn^2+^ ions negatively affects the stability of *SOD1*, leading to increased misfolding of the enzyme (Takashima et al. 2021; Kosmachevskaya, Novikova et al. 2024). This suggests that reduced Hb levels might operate through similar mechanisms as *SOD1* mutations. Additionally, a study involving 3507 men aged 71–93 years who did not have PD found that Hb levels decline with advancing age. Disturbances in iron homeostasis, oxidative stress, and inflammation are shared pathological mechanisms in neurodegenerative diseases that can both directly and indirectly influence Hb's oxygen‐binding affinity, resulting in chronic hypoxia and secondary erythrocytosis (Graham et al. 2014). Hb has the potential to serve as a biomarker for ALS (Leone et al. 2022).

## 5 Conclusion

In summary, our data convincingly reveals a causal association between genetically predicted lower Hb levels and an increased risk of ALS, independent of blood pressure, LDL‐C, total cholesterol levels, BMI, and smoking. These findings imply that dealing with Hb levels may present a potential strategy to reduce the risk of ALS. Further studies should explore the causal relationship in different subgroups, such as ethnic variations, genetic mutations, and disease stages.

## Author Contributions

**Hongmei Luo**: conceptualization, data curation, formal analysis, investigation, writing – original draft, writing – review & editing. **Yujie Yang**: data curation, investigation, formal analysis. **Xiaojian Cao**: methodology, validation, visualization. **Chunchu Deng**: conceptualization, data curation, formal analysis, writing – original draft, writing – review & editing. **Hong Chen**: conceptualization, data curation, resources, funding acquisition, project administration, writing – review & editing. All authors approved the final version.

## Funding

This research was supported by funding from the National Natural Science Foundation of China (Grant No. 82171422).

## Ethics Statement

The data used in this investigation were taken from previously released GWAS. All study participants provided written informed permission to each institutional review board that took part in this research.

## Conflicts of Interest

The authors declare that they have no conflict of interest.

## Supporting information




**Table S1**: brb371152‐sup‐0001‐TableS1.xlsx

**Table S2**: brb371152‐sup‐0002‐TableS2.xlsx

**Figure S1**: brb371152‐sup‐0003‐Figure1.pdf

**Figure S2**: brb371152‐sup‐0004‐Figure2.pdf

**Figure S3**: brb371152‐sup‐0005‐Figure3.pdf

## Data Availability

The data that support the findings of this study are available from the corresponding author upon reasonable request.
