# Supplementary figures and images for: Unveiling the Genetic Association Between Hemoglobin Concentration and Amyotrophic Lateral Sclerosis
Source: Brain Behav. 2025 Dec 31;16(1):e71152. doi: 10.1002/brb3.71152 (PMC12755399; doi:10.1002/brb3.71152)

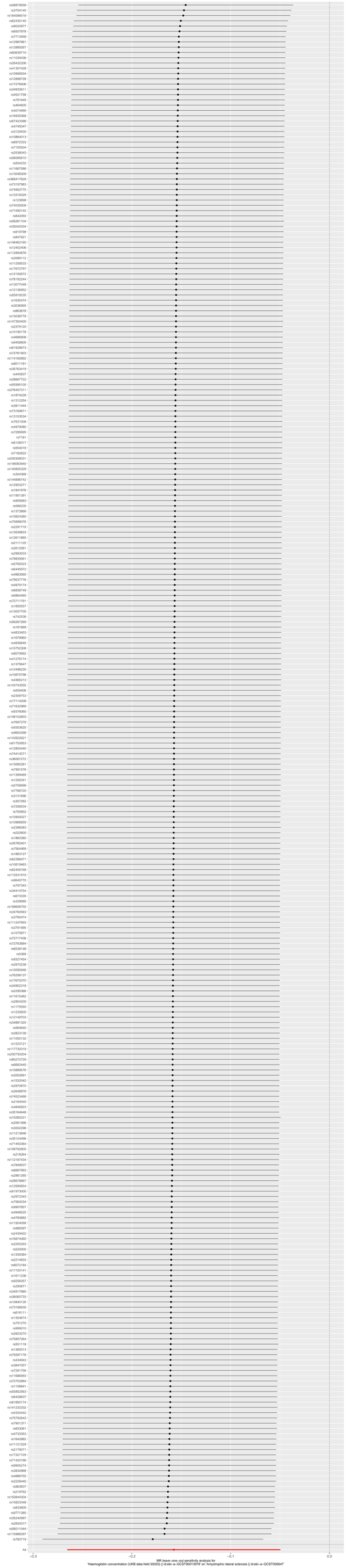

Supplement: Supplementary file 3 — Figure S1: brb371152‐sup‐0003‐Figure1.pdf [file BRB3-16-e71152-s002.pdf]
