# Supplementary material for: Unveiling the Genetic Association Between Hemoglobin Concentration and Amyotrophic Lateral Sclerosis
Source: Brain Behav. 2025 Dec 31;16(1):e71152. doi: 10.1002/brb3.71152 (PMC12755399; doi:10.1002/brb3.71152)
Supplement: Supplementary file 4 — Figure S2: brb371152‐sup‐0004‐Figure2.pdf [file BRB3-16-e71152-s003.pdf]

exposure	outcome	method	b	se	pval	or
Amyotrophic lateral sclerosis	Hemoglobin concentration	MR Egger	2.135721e−02	0.03992409	0.6462018	1.0215869
		Weighted median	8.150947e−05	0.01064686	0.9938917	1.0000815
		Inverse variance weighted	2.766736e−03	0.01597101	0.8624668	1.0027706
		Simple mode	−4.887073e−03	0.01938504	0.8172446	0.9951248
		Weighted mode	1.540744e−02	0.01124255	0.2640856	1.0155267

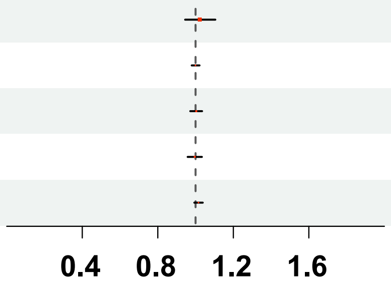

Supplementary Figure 2. Forest plot illustrating the results of the causal effect of ALS on hemoglobin concentration using five MR methods. OR, odds ratio; b, effect size; se, standard error of the effect size; IVW, inverse variance weighted. A p-value < 0.05 was considered statistically significant. OR > 1 indicates a risk factor, while OR < 1 suggests a protective factor.
