# Supplementary material for: Unveiling the Genetic Association Between Hemoglobin Concentration and Amyotrophic Lateral Sclerosis
Source: Brain Behav. 2025 Dec 31;16(1):e71152. doi: 10.1002/brb3.71152 (PMC12755399; doi:10.1002/brb3.71152)
Supplement: Supplementary file 5 — Figure S3: brb371152‐sup‐0005‐Figure3.pdf [file BRB3-16-e71152-s001.pdf]

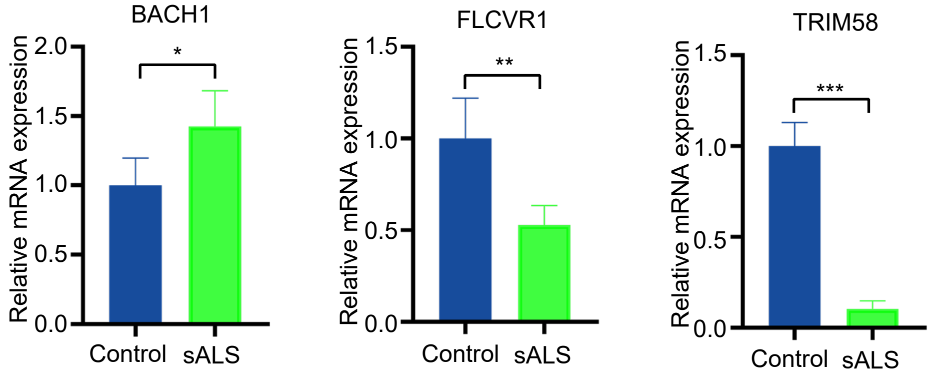

Supplementary Figure 3: qRT-PCR analysis showing the expression levels of *BACH1*, *FLVCR1*, and *TRIM58* in Control versus sALS MNs, with error bars indicating the mean ± SD. Statistical significance is denoted by asterisks: *$p < 0.05$, **$p < 0.01$ and ***$p < 0.001$, while ns represents not significant. Asterisks indicate significant differences between groups as determined by the t-test.
